# Analysis of mitochondrial organization and function in the *Drosophila* blastoderm embryo

**DOI:** 10.1038/s41598-017-05679-1

**Published:** 2017-07-14

**Authors:** Sayali Chowdhary, Darshika Tomer, Dnyanesh Dubal, Devashree Sambre, Richa Rikhy

**Affiliations:** Biology, Indian Institute of Science Education and Research, Homi Bhabha Road, Pashan, Pune, 411008 India

## Abstract

Mitochondria are inherited maternally as globular and immature organelles in metazoan embryos. We have used the *Drosophila* blastoderm embryo to characterize their morphology, distribution and functions in embryogenesis. We find that mitochondria are relatively small, dispersed and distinctly distributed along the apico-basal axis in proximity to microtubules by motor protein transport. Live imaging, photobleaching and photoactivation analyses of mitochondrially targeted GFP show that they are mobile in the apico-basal axis along microtubules and are immobile in the lateral plane thereby associating with one syncytial cell. Photoactivated mitochondria distribute equally to daughter cells across the division cycles. ATP depletion by pharmacological and genetic inhibition of the mitochondrial electron transport chain (ETC) activates AMPK and decreases syncytial metaphase furrow extension. In summary, we show that small and dispersed mitochondria of the *Drosophila* blastoderm embryo localize by microtubule transport and provide ATP locally for the fast syncytial division cycles. Our study opens the possibility of use of *Drosophila* embryogenesis as a model system to study the impact of maternal mutations in mitochondrial morphology and metabolism on embryo patterning and differentiation.

## Introduction

Subcellular organelles are transferred to the developing oocyte from surrounding sister germ cells in vertebrates such as mouse and invertebrates such as *Drosophila* and are important for their differentiation. The metazoan embryo attains subcellular organelles such as the endoplasmic reticulum (ER), Golgi complex and mitochondria by maternal cytoplasmic inheritance. Mitochondria are eliminated pre-fertilization in *Drosophila* sperm^1^ and post fertilization in the zygote in *Caenorhabditis elegans* ^2^ and primates^3^. Mutations in the mitochondrial genome are responsible for maternal transmission of a plethora of mitochondrial diseases in offsprings. Mitochondria occur in a small, globular state and in limited numbers in stem cells and early embryos. They are transformed from a nascent immature structure in stem cells to a more tubular, higher ATP generating architecture during differentiation^4^. Even though early human embryos have relatively small mitochondria^5, 6^, they show activation of the electron transport chain (ETC) resulting from calcium waves during fertilization^7–10^.

Mitochondria function in discrete locations in close association with other organelles such as the ER and the cytoskeleton to provide various functions such as ATP generation and calcium buffering locally. The ER-mitochondria encounter sites show an accumulation of mitochondrial fission machinery and are often found adjacent to mitochondrial nucleoid replication to allow appropriate distribution of nucleoids during fission^11, 12^. Mitochondria are trafficked to the neuronal synapse on microtubules^13^ and this is essential for meeting local energy demands in synaptic function during increased activity and for calcium homeostasis^14–16^. Mitochondria bind via adaptors to microtubule based motor proteins such as Kinesin and Dynein^17–21^. Mutations in the mitochondrial Kinesin linker protein, Miro lead to increased accumulation of mitochondria in cell body and motor neuron diseases^22^. Mitochondria also interact with F-actin at mitochondrial-ER contact sites for mitochondrial fission^12^. Thus mitochondria closely associate with cytoskeletal components, which drive their trafficking to desired locations based on local ATP demands. Taken together, mitochondrial morphology, distribution and transport are also likely to be important for cellular function in metazoan embryos but this has not been explored in detail.

**Figure 1:**
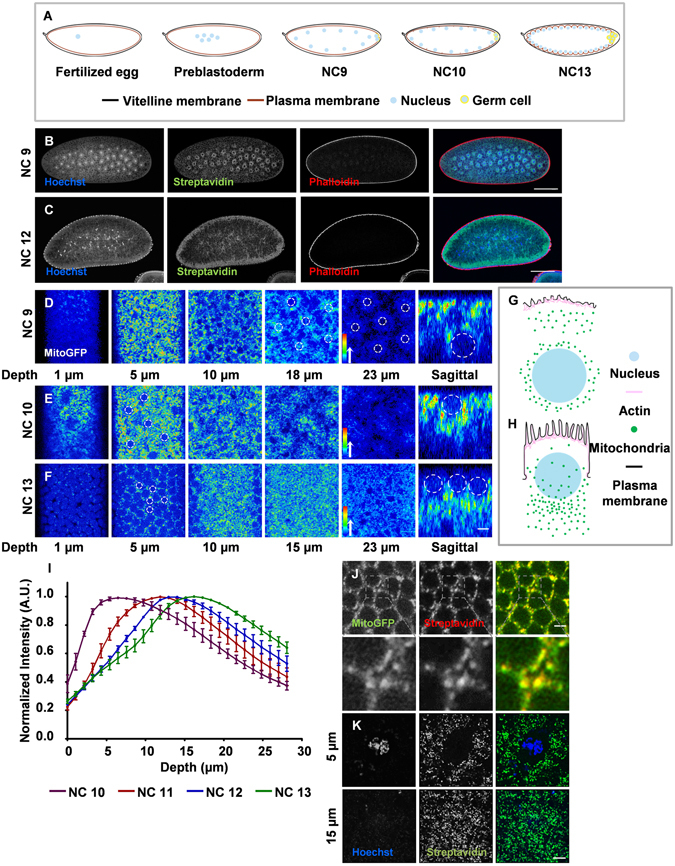
Mitochondrial distribution in syncytial *Drosophila* cycles. (**A**) Schematic showing nuclear cycles of *Drosophila* syncytial embryo. (**B**,**C**) Mitochondrial distribution in preblastoderm and blastoderm embryos. Mitochondria (Streptavidin; green) are seen around nuclei (Hoechst; blue) (Phalloidin; red) in the preblastoderm NC9 at approximately 23 µm depth from the surface (**B**). Mitochondria are enriched around nuclei at the cortex in blastoderm NC12 (**C**). (**D**–**I**) Mitochondrial intensity increases with depth during syncytial cycles. Optical sections at depths shown were obtained from live imaging of embryos expressing Mito-GFP through the syncytial cycles. Mito-GFP is seen cortically and around nuclei (white dashed lines) at 23 µm depth (**D**) and in sagittal section at NC9. Mitochondria distribute around nuclei as they arrive at 5 µm in NC10 (**E**) and localize below the nuclei (**E**, sagittal). Mitochondrial intensity is higher below the nuclei at 10–15 µm depth in NC13 (**F**) and also seen in sagittal section (**F**). Schematic of mitochondrial distribution in sagittal view as seen in preblastoderm (**G**) and syncytial blastoderm embryos (**H**). Quantification of mean mitochondrial fluorescence intensity across depth from apical to basal region (**I**). n = 3 embryos/NC, 15, 30, 60 and 120 syncytial cells were quantified for NC10 (Purple), 11 (Red), 12 (Blue) and 13 (Green) respectively. Error bars show SEM. Rainbow scale represents pseudocolored intensities. (**J**) Streptavidin colocalizes with Mito-GFP. Mitochondria labelled with Mito-GFP (green) at NC13 are stained with fluorescent conjugated Streptavidin (red). (**K**) Mitochondria are small and dispersed in the syncytial embryo. High resolution imaging of dispersed mitochondria in NC11, Streptavidin (green); Nucleus (blue) at section through nucleus (5 µm) and basal section (15 µm). Scale bar B,C: 100 µm, D-F: 10 µm, J: 5 µm, K: 2 µm.

The analysis of distribution and dynamics of mitochondria with respect to time in *Drosophila* embryogenesis has not been carried out so far and will allow a correlation of mitochondrial attributes with various cellular events occurring during embryogenesis. *Drosophila* embryogenesis starts as a syncytium with nuclear division cycles 1–9 taking place within the interior of the embryo followed by migration of nuclei to the cortex in nuclear cycle (NC) 10 forming the syncytial blastoderm. NC11–13 occur at the periphery in the syncytial blastoderm (Fig. 1A). Plasma membrane extension in NC14 encloses approximately 6000 nuclei in a process called cellularization to form complete cells. The ER and the Golgi complex are enriched peripherally in the syncytial embryo. Despite the absence of plasma membrane boundaries, the syncytial embryo exhibits compartmentalization of organelles and plasma membrane to a region around the nucleus. Proteins in the ER lumen show decreased diffusion between adjacent nucleo-cytoplasmic regions and this restriction is dependent on microtubule organization^23^. Secretion from the Golgi complex shows localized delivery of proteins to the plasma membrane above it. The continuous plasma membrane above each nucleus is compartmentalized and organized in an epithelial-like manner^23, 24^. Hence we refer to each nucleo-cytoplasmic domain containing its organelles and plasma membrane as a “syncytial cell”. Mitochondria have been observed earlier in electron microscopy studies in *Drosophila* embryos^25^. We have standardized methods to view and quantify the distribution and function of mitochondria in living and fixed *Drosophila* embryos. We find that mitochondria do not move between neighbouring syncytial cells and are distributed equally to daughter syncytial cells. Their density along the apico-basal axis in the syncytial *Drosophila* embryo depends upon appropriate microtubule based transport and their functionality is important for generation of local ATP, which drives the syncytial division cycles of the blastoderm embryo.

## Results

### Mitochondria are dispersed and enriched at the cortex in the syncytial *Drosophila* embryo

Subcellular organelles such as ER and Golgi complex are present at the peripheral cortex up to approximately 20 µm beneath the plasma membrane of the *Drosophila* pre-blastoderm and blastoderm embryo. When nuclei and centrosomes arrive at the periphery, the ER membrane clusters and surrounds each nucleus^23^. We used *nanos*-Gal4 to drive the expression of mitochondrially targeted GFP (Mito-GFP) during oogenesis to visualize mitochondria in early *Drosophila* embryos. Mito-GFP contains the mitochondrial localization sequence from cytochrome oxidase VIII which targets GFP to the mitochondria and has been used to study mitochondrial distribution in the developing oocyte^26^. Unlike ER^23^, mitochondria were seen around deep pre-blastoderm nuclei (20–23 µm from the plasma membrane), even before their arrival to the cortex (Fig. 1,B,D). Mitochondria were also present in cortical regions of the blastoderm embryo (Fig. 1B,D,G) and they distributed around nuclei on arrival at the cortex in NC10 of the syncytial blastoderm (Fig. 1E). Mitochondria congregated around nuclei at the cortex in the syncytial division cycles NC10-13 and asymmetrically distributed in the apico-basal plane of syncytial blastoderm cells (Fig. 1E–H). We quantified the mean mitochondrial intensity in optical stacks across depth in the Z axis from apical to basal sections using Mito-GFP in NC10-13 of syncytial embryos from movies obtained from living embryos. Mitochondria were sparsely present in apical regions and their density increased towards the basal regions in syncytial blastoderm cells (Fig. 1I). The basal localization of mitochondria became more pronounced with each NC. The highest mitochondrial intensity was observed at 7 µm, 11 µm, 13 µm and 14 µm depths for NC10, 11, 12 and 13 respectively (Fig. 1I). This increase in the depth of enrichment of mitochondria across syncytial cycles correlates with previous observations of increase in metaphase furrow lengths^27^ and increased tubulin spread in depth from the cortex across syncytial division cycles suggesting that the distribution is likely to be regulated by cytoskeletal organization and transport differences across cycles and may be important for local supply of ATP.

We stained mitochondria with fluorescently labelled Streptavidin, which colocalized with Mito-GFP (Fig. 1J). Streptavidin is known to colocalize with mitochondria because of an enrichment of biotinylated proteins in the mitochondrial matrix in specific tissues^28^. We found that it is a good marker for mitochondria in the syncytial embryo. Using high-resolution confocal microscopy with Airyscan (Carl Zeiss, LSM 800), we observed that mitochondria were punctate and dispersed in optical planes around the DNA (5 μm) and in basal sections (15 μm) in the syncytial embryo in NC11 (Fig. 1K). This was in contrast to the more elaborate mitochondrial network that has been observed in follicle cells, nurse cells and neuroblasts from *Drosophila* tissues^18, 29, 30^, but almost similar to discrete small mitochondria seen in vertebrate embryo^6, 7^.

We further compared mitochondrial distribution to the ER and Golgi complex in syncytial cells. We used *nanos*-Gal4 to drive the expression of both Mito-GFP and ER resident KDEL-RFP in syncytial cells. ER tubules form a meshwork around nuclei during syncytial cycles^23^. Mitochondria were present within the ER meshwork (Fig. S1A). Mitochondrial fission occurs at ER mitochondria contact sites^11, 12^. To image mitochondria and Golgi together, we used *nanos*-Gal4 to drive the expression of Mito-GFP and galactosyl transferase (GalT)-RFP in embryos. Mitochondria were distributed like the Golgi complex as punctate structures in the syncytial *Drosophila* embryo and their distribution along the apical to basal axis across the embryo was also similar. Like mitochondria, Golgi bodies displayed basal abundance (Fig. S1B) but the mitochondrial density was much higher as compared to the Golgi complex. It is likely that an appropriate concentration of both mitochondria and Golgi complex is maintained by directed transport in the basal direction during syncytial blastoderm stage. The Golgi complex is transported in the apical direction during cellularization using microtubules based motors^31, 32^.

In summary, we found that mitochondria are small, dispersed and organized as punctate structures with increase in density basally in syncytial cells of the *Drosophila* embryo. This discrete distribution points to regulated transport of mitochondria along the apico-basal axis and may be driven by microtubule-based transport.

### Mitochondria show restricted lateral movement and distribute equally to daughter cells in the syncytial *Drosophila* embryo

**Figure 2:**
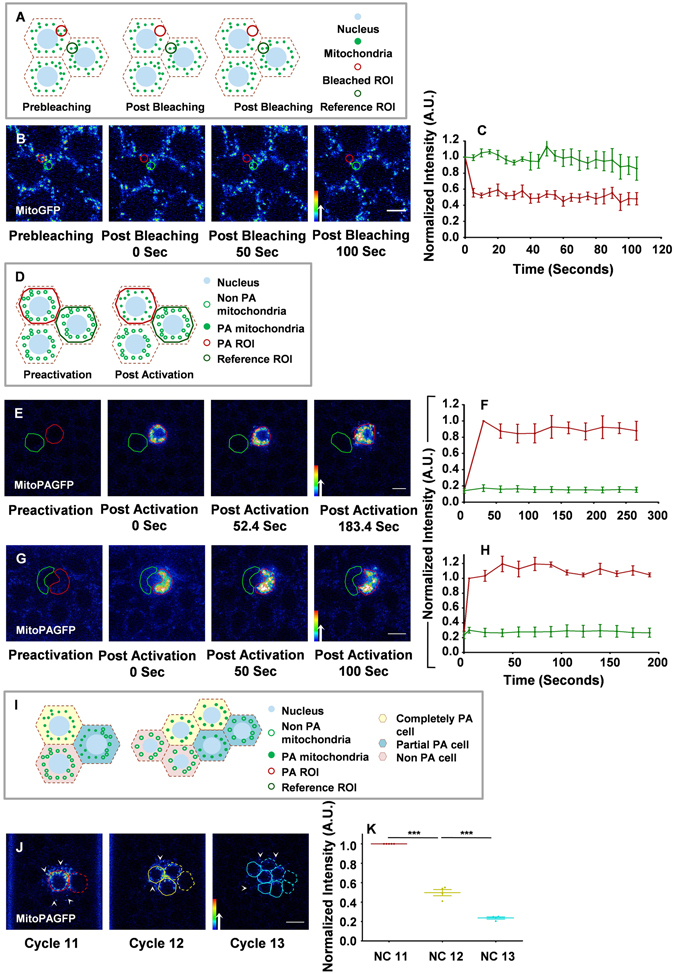
Mitochondria are immobile in the lateral plane in the syncytial *Drosophila* embryo. (**A**–**C**) Mito-GFP FLIP shows restricted loss of fluorescence. FLIP schematic with photobleach ROI (red) and reference ROI (green) (**A**). Continuous photobleaching of Mito-GFP is done in an ROI of 4 μm^2^ (red) in interphase of the NC13 (**B**). Fluorescence in the neighbouring syncytial cell ROI (green) does not deplete with time. Normalized mean intensity of bleached (red) and non-bleached (green) ROI is plotted with respect to time (**C**). (**D**–**H**) Photoactivated mitochondria are restricted in syncytial cells. Non-photoactivated and photoactivated mitochondria are represented as hollow and filled green circles respectively in a schematic of the photoactivation experiment (**D**). Mito-PAGFP is activated in one syncytial cell (red), fluorescence is monitored in neighbouring cell (green), which shows no gain in fluorescence in the given time (**E**) in interphase of NC12. Normalized mean fluorescence intensity of activated (red) and neighbouring (green) syncytial cell is plotted with respect to time (**F**). Mito-PAGFP is activated in a part of syncytial cell (red) and neighbouring region (green) from the same cell is monitored for fluorescence intensity changes (**G**). Neighbouring region does not have an increase in fluorescence. Normalized mean fluorescence intensity of activated (red) and neighbouring (green) region is plotted against time (**H**). (**I**–**K**) Photoactivated mitochondria are distributed equally to daughter syncytial cells. Schematic of photoactivated mitochondria distribution from mother to daughter syncytial cells (**I**). Mother cell and respective daughter cells are represented by the same colour. Completely photoactivated cell (yellow), partially photoactivated cell (green) (also see Fig. S2), non-photoactivated cell (orange) (**I**). Mito-PAGFP is activated in the ROI comprising one syncytial cell in NC11 (red) interphase and partially in the neighbours (white arrows) and mean intensity is measured in daughter syncytial cells of NC12 (yellow, solid line, originating from the red syncytial cell) and 13 (cyan, solid line). (**J**): Mean intensity in NC11–13 is normalized to corresponding NC11 intensities (**K**). (***P ≤ 0.001, Student’s t-test). n = 3 embryos for C, F and H. 4 embryos for K. Error bars represent SEM. Scale bar: 5 µm. Rainbow scale represents pseudocolored intensities.

The ER and the Golgi complex are compartmentalized to one syncytial cell in the *Drosophila* embryo. Proteins in the ER lumen diffuse slowly between adjacent syncytial blastoderm cells. The punctate Golgi complexes are closely associated with ER and do not travel to the neighbouring syncytial cell^23^. Trafficking through the ER and Golgi complex is important for local delivery of cargo to the plasma membrane associated with an individual syncytial cell. Like these organelles, mitochondrial function is also likely to be essential locally. We therefore asked whether mitochondria also show compartmentalization in syncytial cells and whether their function is essential for progressing the syncytial division cycles. Using continuous photobleaching of Mito-GFP, we monitored fluorescence loss in neighbouring syncytial cells to assess if mitochondria were moving between them in interphase of the syncytial cycle (materials and methods) (Fig. 2A). We continuously photobleached a region of interest (ROI, red) and observed no reduction in Mito-GFP fluorescence on monitoring an ROI in neighbouring syncytial cells (green) showing that mitochondrial movement is restricted to individual syncytial cells in the embryo over the time scale of 100 s within interphase of the same syncytial cycle (Fig. 2B,C) when the plasma membrane length is known to be the shortest^27^. No depletion of signal from the neighbouring syncytial cell was observed in photobleaching experiments in previous studies over a similar time scale for the Golgi Complex, ER and plasma membrane^23, 24^.

We used photoactivation to highlight a specific mitochondrial population more precisely in syncytial cells and monitored their distribution within and across adjacent syncytial cells over time. The mitochondrial photoactivatable GFP (Mito-PAGFP)^33^ construct was made by fusion with a mitochondrial localisation signal of cytochrome oxidase VIII. Transgenic flies with Mito-PAGFP were combined with *nanos*-Gal4 to express it in embryos. The Mito-PAGFP signal overlapped completely with fluorescent Streptavidin in fixed embryos showing that it targets appropriately to mitochondria (Fig. S2A). A similar Mito-PAGFP construct has been used to label a specific mitochondrial population in order to assess its migration to specific location in mammalian cells^34^.

We photoactivated Mito-PAGFP fluorescence (materials and methods, Fig. 2D) in an entire syncytial cell in interphase and monitored neighbouring cells for appearance of fluorescent mitochondria (Fig. 2E and Movie S1). This also allowed us to follow mitochondrial movements for a longer duration in interphase of the syncytial cycle. The fluorescence was at its maximum in the photoactivated cell in the first 30 s and did not increase in the neighbouring syncytial cells confirming that there was no exchange of mitochondria between them over the time scale of more than 270 s within the same syncytial cycle (Fig. 2E,F and Movie S1).

We then photoactivated mitochondria in approximately half the syncytial cell and followed the non-activated region of the same cell to track mitochondrial movement within a syncytial cell (Fig. 2G). Whereas the fluorescence increased to its maximum in the photoactivated region within 10 s, neither the non-activated region of the same syncytial cell nor the neighbouring syncytial cell gained any mitochondrial fluorescence in 180 s (Fig. 2G,H). This confirmed that the mitochondria in syncytial cells were considerably stationary in the lateral plane in the given interphase time scale and were not free to travel to neighbouring syncytial cells. This was in contrast to the ER where lumenal proteins show free diffusion of within the entire network in one syncytial cell^23^.

Finally we followed and quantified photoactivated mitochondria in one syncytial cell across 2 division cycles in order to study their distribution between daughter cells. We photoactivated a syncytial cell completely (Fig. 2I, yellow cell, Fig. 2J) or partially (Fig. 2I, green cell, Fig. S2B). We found that the intensity of the photoactivated mitochondrial pool was halved in daughter syncytial cells from one division cycle to the next from NC11 to 13 (Fig. 2I–K and Movie S2). When a cell was partially photoactivated on one side of the spindle, the mitochondria were asymmetrically distributed to the daughter cell formed on the same side (Fig. S2B,C and I). This showed that mitochondrial movement is restricted and they are systematically distributed between mother syncytial cells and daughter syncytial cells in a lineage specific manner.

### Mitochondria move in the apico-basal axis to maintain constant apical numbers in successive syncytial division cycles in the *Drosophila* embryo

**Figure 3:**
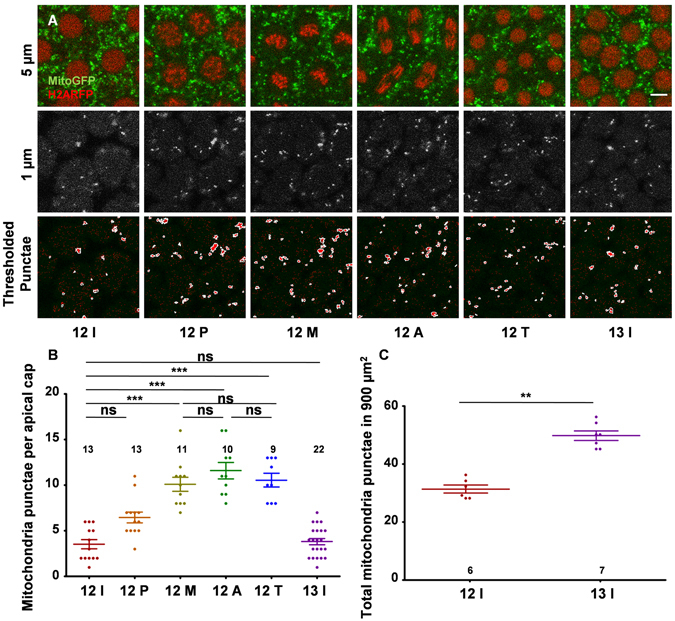
Apical mitochondrial punctae numbers remain constant across the syncytial division cycle. (**A**) Apical mitochondria increase during metaphase of the syncytial cycle. Live imaging of embryos expressing Mito-GFP (green) and Histone2A-RFP (red) is shown across the NC12 at 1 µm and 5 µm depths (12I: interphase NC12, 12 P: prophase, 12 M: metaphase 12 A: anaphase 12 T: telophase 13I: interphase NC13). At 1 µm depth, mitochondria are present as discrete countable structures (2nd panel). Using ImageJ, distinct punctae are thresholded, marked and quantified (3rd panel). Scale bar A: 5 µm. (**B**,**C**) Quantification of mitochondria across syncytial cycles. Number of distinctly separable mitochondrial puncta per syncytial cell significantly increases in metaphase and the number is conserved between 12I and 13I (**B**) (N = 4 embryos, 13, 13, 11, 10, 9, 22 syncytial cells for 12I, 12 P, 12 M, 12 A, 12 T and 13I respectively. ns–not significant; ***P ≤ 0.001, one way Kruskal Wallis test). Total number of mitochondria in 900 μm^2^ area are compared between 12I and 13I (**C**) (n = 6 and 7 embryos; each point is an average from 20 and 50 syncytial cells for 12I and 13 I respectively, **P ≤ 0.01, two tailed Mann-Whitney test).

We further quantified mitochondrial abundance in different stages of the syncytial cycle and observed that the numbers of mitochondria increased in apical sections (Fig. 3A, 1 μm) in a stage specific manner in syncytial cycles. Discrete mitochondria were visible in the apical sections above the nucleus (Fig. 3A, 5 μm) of the syncytial *Drosophila* embryo (Movies S3 and S4). In order to assess the mitochondrial distribution in a semi-quantitative manner in syncytial cells across nuclear division cycles we counted the number of mitochondrial punctae present in these apical sections (Fig. 3A, 1 μm) across NC12 in Mito-GFP expressing embryos. An intensity-based threshold was applied to mark and quantify mitochondrial punctae (Fig. 3A, thresholded punctae) (materials and methods). On counting the numbers of optically resolvable mitochondrial punctae in individual syncytial cells in interphase (12I), prophase (12 P), metaphase (12 M), anaphase (12 A) and telophase (12 T) of NC12 and interphase of NC13 (13I), a significant increase was observed in 12 M, 12 A and 12 T (Fig. 3A,B and Movie S3). Interestingly, the numbers of these punctae per syncytial cell were similar in 12I and 13I (Fig. 3B). Likewise, we also found that mitochondrial punctae approximately doubled in a given apical area (900 μm^2^) in 13I where the number of syncytial cells was doubled as compared to 12I (Fig. 3A, 1 μm, C). This increase in apical mitochondria in metaphase and anaphase of the syncytial cycle is likely to be responsible for maintaining a constant mitochondrial density per syncytial cell across syncytial cycles. Since mitochondria were found to be compartmentalized to one syncytial cell and did not move significantly in between adjacent syncytial cells (Fig. 2), this apical increase in mitochondrial intensity could be due to their trafficking from basal sections to populate apical sections during the syncytial division cycle.

### Microtubules regulate mitochondrial content in the apical region of the syncytial *Drosophila* embryo

**Figure 4:**
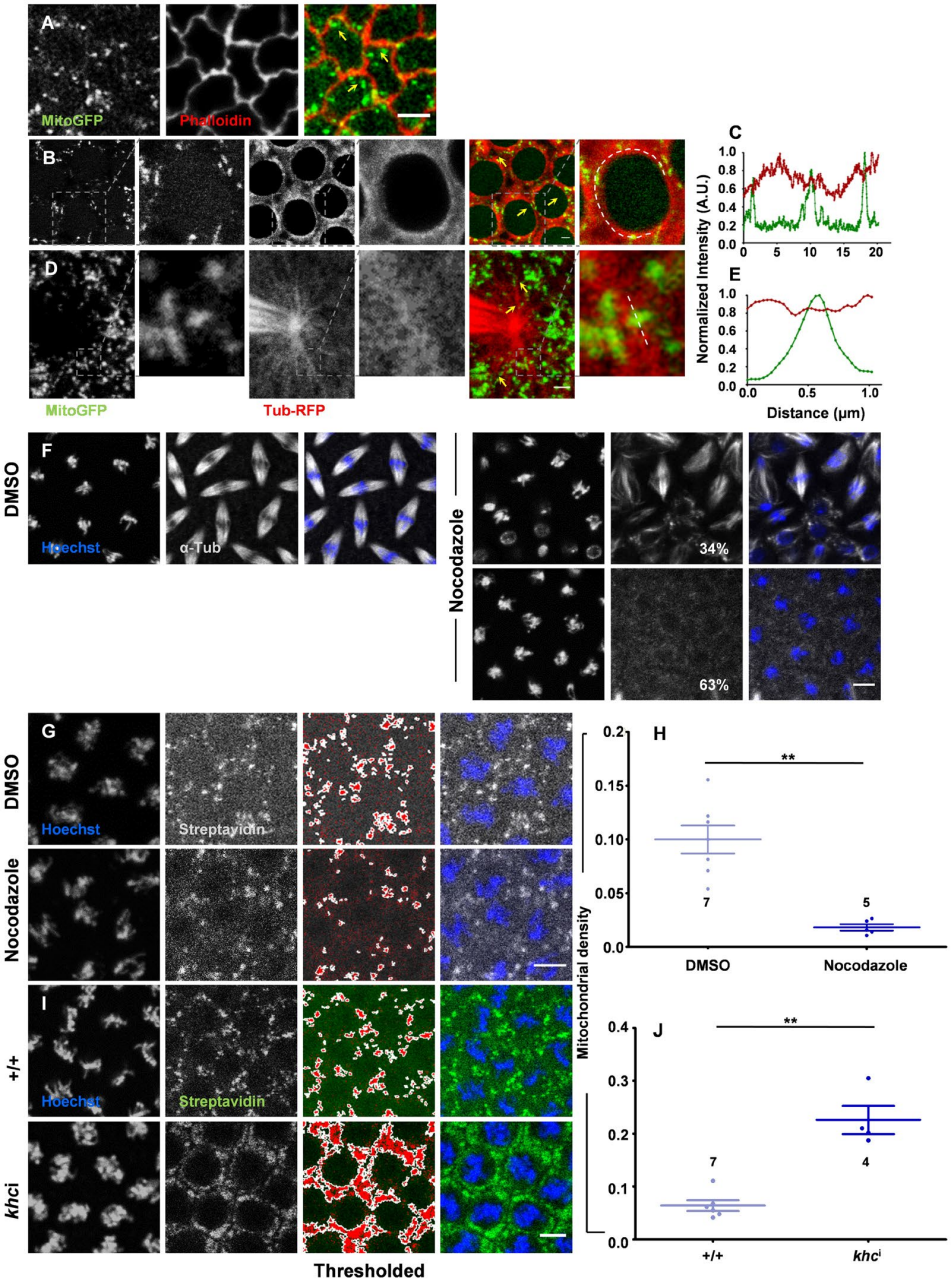
Microtubules regulate mitochondrial density in the apical region in the syncytial *Drosophila* embryo. (**A**) Mitochondria do not colocalize with cortical actin. Mito-GFP (green) and fluorescence tagged Phalloidin (red) do not colocalize. (**B**–**E**) Mitochondria are present in proximity to microtubules in the syncytial cycles. Mito-GFP (green) shows partial overlap with Tub-RFP (red) (**B**,**D**). Mitochondria are lined along perinuclear microtubules in prophase (**B**, zoomed selection to the right) and as seen in the intensity line profile (**C**) and along astral microtubule filaments (**D**, zoomed in images to the right) and intensity profile shown in E. White lines on merged zoomed in images (**B**,**D**) represent region shown in line profiles (**C**,**E**). (**F**–**H**) Nocodazole treatment decreases apical mitochondrial intensity in syncytial cells. Nocodazole treatment leads to partial (34%) or complete (63%) disruption of metaphase spindles (tubulin’ grey) in syncytial embryos compared to DMSO control. Mitochondria (grey) are sparser in Nocodazole treated embryos as compared to control (**G**). The mitochondrial fluorescence is thresholded, marked and the mitochondrial density is quantified (**H**). (n = 7, 4 embryos and 250, 150 syncytial cells are averaged for control, nocodazole treatment respectively, **P ≤ 0.01, two tailed Mann–Whitney test). (**I**–**J**) *khc* ^i^ expressing embryos show an increase in mitochondrial density (green) in apicolateral regions of syncytial cells. (n = 7, 4 embryos, each data point is obtained as an average from 280 and 150 total syncytial cells for control and *khc* ^i^ respectively, **P ≤ 0.01, two tailed Mann-Whitney test). Scale bar: A, F, G and I: 5 µm, B, D: 2 µm.

Mitochondria travel to distinct regions in the eukaryotic cells using the actin and tubulin cytoskeleton^35–37^. In syncytial *Drosophila* cells the actin network is closely apposed to the plasma membrane. Double labelled imaging of an F-actin marker, Phalloidin with Mito-GFP, did not show an appreciable overlap between actin and mitochondria (Fig. 4A). Mitochondria were present internally in proximity to the tubulin network in perinuclear regions (Fig. 4B,C). Mitochondria overlapped with perinuclear microtubules (Fig. 4B,C) and were also found adjacent to centrioles juxtaposed with astral microtubules (Fig. 4D,E).

In order to assess if the tubulin network regulated the apico-basal trafficking of mitochondria, we depolymerized microtubules in embryos using nocodazole and quantified the mitochondrial density in apical sections around the nucleus (materials and methods). As expected nocodazole disrupted the microtubule spindles partially (Fig. 4F, nocodazole, top panel) or completely (Fig. 4F, nocodazole bottom panel) in syncytial embryos as compared to intact spindles observed in DMSO treated controls (Fig. 4F, DMSO). Streptavidin staining was used to assess mitochondrial density (Fig. 4G) and a significant decrease in mitochondrial density was found in the apico-lateral regions around the spindle in nocodazole treated embryos compared to controls (Fig. 4H). Thus an intact microtubule network is essential for distributing the appropriate mitochondrial content in the apical regions of syncytial cells.

Centrosomes are present apically and polymerizing plus ends are oriented basally in syncytial cells of the *Drosophila* embryo^38^. We used RNAi against the heavy chain subunit of the plus ended motor Kinesin (*khc* ^i^) to deplete mitochondrial trafficking in basal regions of the syncytial cells and quantified the mitochondrial density in apical sections. We found a significant increase in the mitochondrial density in apical sections around the nucleus in *khc* ^i^ (Fig. 4I,J) as compared to control embryos (Fig. 4I,J). This is because of lack of mitochondrial transport to basal sections along the microtubule network and an unbalanced mitochondrial transport towards the minus end. This increase in mitochondrial density shows that Kinesin is responsible for trafficking mitochondria to basal sections in the syncytial *Drosophila* embryo.

In summary mitochondrial distribution along the apico-basal axis in the syncytial *Drosophila* embryo is due to association and trafficking on microtubules. This is also a likely mechanism for maintaining the restricted movement of mitochondria and compartmentalization in the ER lumen in syncytial embryos^23^.

### Depletion of the mitochondrial ETC reduces ATP and increases pAMPK in syncytial *Drosophila* embryos

**Figure 5:**
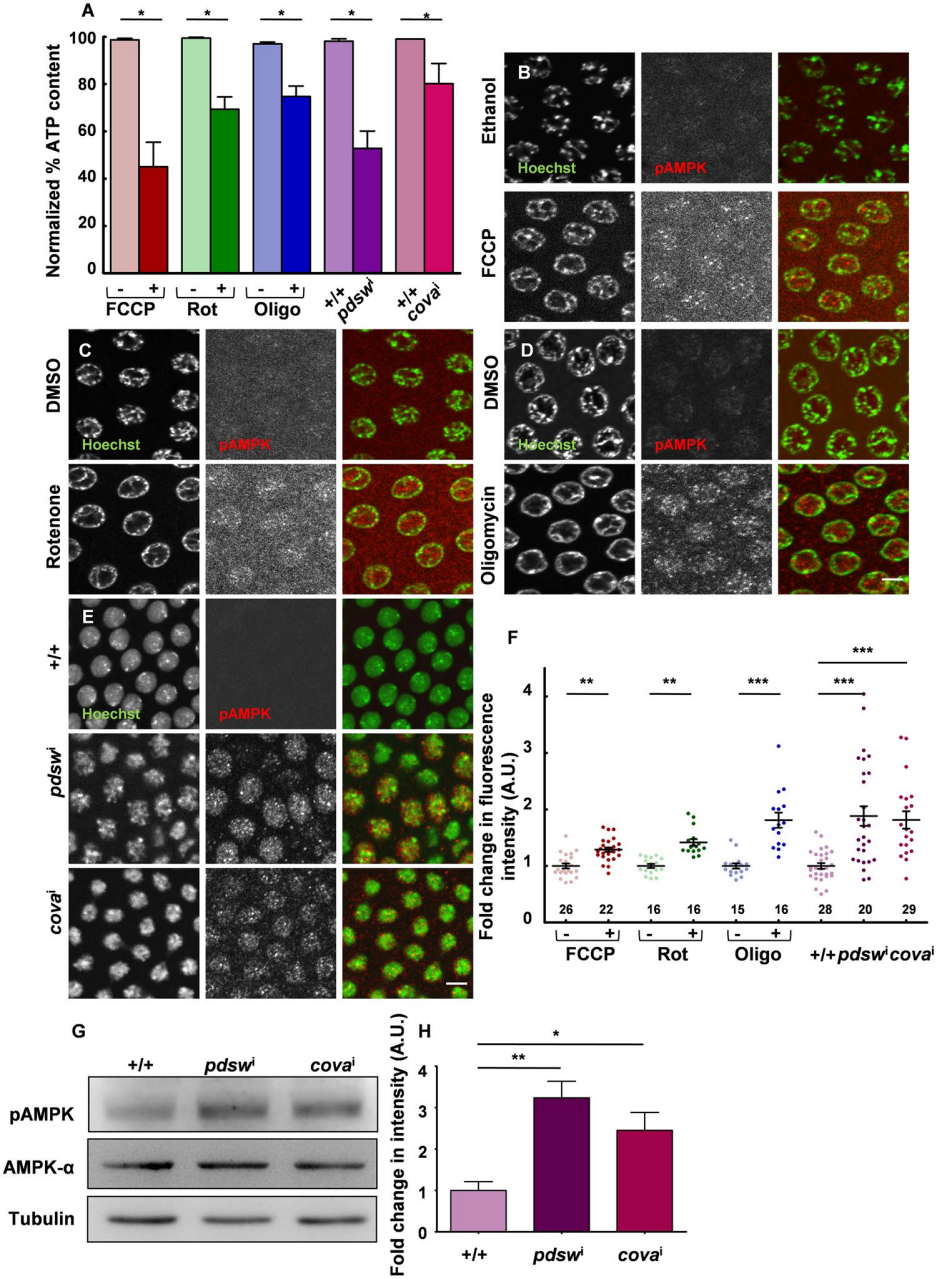
ETC depletion reduces ATP and increases pAMPK in syncytial *Drosophila* embryos. (**A**) Decrease in ATP due to ETC inhibition. The ATP concentration determined by a luciferase based assay is normalized to amount of protein from embryos in each sample and represented as a percentage of the corresponding control. FCCP (red), Rotenone (green) and Oligomycin (blue) treated embryos and *pdsw* ^i^ (purple) and *cova* ^i^ (pink) mutant embryos show a significant reduction in ATP. (*P ≤ 0.05, n = 9, N = 3, one tailed Mann–Whitney test). (**B**–**F**) Genetic and pharmacological inhibition of the ETC shows increased pAMPK in syncytial embryos. Elevated pAMPK (red in merged) signal is seen in FCCP (**B**), Rotenone (**C**) and Oligomycin (**D**) treated embryos compared to respective controls imaged at the same settings. *pdsw* ^i^ and *cova* ^i^ (**E**) show increased pAMPK signal compared to control embryos. Normalized fluorescence intensity of pAMPK in embryos treated with FCCP (red), Rotenone (green) and Oligomycin (blue) and *pdsw* ^i^ (purple) and *cova* ^i^ (pink) compared to their respective controls represented by lighter shades (**F**). Numbers and data points represent embryos analysed. (**P ≤ 0.01; ***P ≤ 0.001, two tailed Mann-Whitney test.). n = 22, 26 embryos for control and FCCP; 16,16 embryos for control and Rotenone; 16, 15 embryos for control and Oligomycin; 29, 28 and 20 embryos for control, *pdsw* ^i^ and *cova* ^i^ respectively (**H**). On an average 21 syncytial cells per embryo were measured. N: FCCP-3, Rotenone and Oligomycin-4, *pdsw* ^i^ and *cova* ^i^-3. Scale bar: 5 μm. (**G**–**H**) Western blot of pAMPK and total AMPK in WT, *pdsw* ^i^ and *cova* ^i^ expressing embryos. Band intensity of pAMPK in normalized with total AMPK within each lane and represented as fold change with respect to WT (**H**). The full length blots are shown in Fig. S3F. Error bars represent SEM, N = 3, (**P ≤ 0.01; *P ≤ 0.05, Student’s t test).

The discrete distribution of mitochondria in the syncytial *Drosophila* cells is presumably important for generating local ATP for the syncytial division cycle. Mitochondrial ATP generated by ETC activity is an important source of energy in different metazoan embryos^7–10, 39^. Small and dispersed mitochondria, like the ones seen in the syncytial *Drosophila* embryo (Fig. 1), are likely to be relatively poor ATP generators^40, 41^. However, *Drosophila* syncytial embryos metabolize amino acids for energy^42^ and may depend on the ETC activity for ATP. We assessed if cytoplasmic ATP could be depleted in syncytial *Drosophila* embryos by pharmacological and genetic reduction of the mitochondrial ETC activity. In order to disrupt ETC activity we used an acute treatment protocol using pharmacological inhibitors against various ETC complexes. FCCP (Carbonyl cyanide-*4*-(trifluoromethoxy) phenylhydrazone) is an ionophore which transports protons from the mitochondrial inner membrane to the mitochondrial matrix and disrupts the potential difference thereby reducing ATP synthesis^43^. Rotenone specifically inhibits Complex I of the ETC by preventing electron transfer to Coenzyme Q^44^. Oligomycin is an inhibitor of the F0 subunit of the ATP synthase complex and reduces ATP output by ETC^45^. We permeabilized syncytial embryos using the limonene-heptane combination^46^ and treated them with FCCP, Rotenone and Oligomycin (materials and methods). We quantified ATP levels following drug treatment using luciferase based ATP determination assay and found a consistent and significant drop in ATP levels in embryos treated with FCCP, Rotenone and Oligomycin as compared to the corresponding controls (Fig. 5A). We used this assay to determine if the ATP content was reduced by genetically depleting the ETC components PDSW subunit of NADH dehydrogenase complex (complex I, *pdsw*) and cytochrome C oxidase subunit 5a (complex IV, *cova*) using RNAi (*pdsw* ^i^ and *cova* ^i^) driven by *nanos*-Gal4. *pdsw* ^i^ and *cova* ^i^ embryos showed a decrease in ATP as compared to controls similar to the treatment with ETC blocking pharmacological agents (Fig. 5A).

We tested if phosphorylated AMPK (pAMPK) was increased in embryos depleted of ATP on treatment with mitochondrial drugs by immunostaining and western blotting. In metabolically active cells, ATP is continuously hydrolysed and converted to ADP and AMP. In eukaryotic cells a highly conserved protein AMP kinase (AMPK) acts as a metabolic sensor, which is phosphorylated in low ATP conditions with AMP accumulation^47^. Control embryos exhibited predominantly nuclear pAMPK localization (Fig. S3A,B) while total AMPK-α showed a uniform distribution and was both nuclear as well as cytoplasmic (Fig. S3B). In prophase, pAMPK immunostaining colocalized with the whole nucleus (Fig. S3A, top panel). A number of studies show nuclear and centrosomal localization of pAMPK^48–50^. We found a significant increase in pAMPK levels in the nucleus and cytoplasm upon acute inhibition of the ETC using pharmaceutical drugs in syncytial embryos (Fig. 5B–D). There was an average of 1.3, 1.4 and 1.8 fold increase in pAMPK immunostaining intensity in FCCP, Rotenone and Oligomycin treated embryos respectively compared to their independent controls (Fig. 5F). We also assessed if a similar change was seen on genetic abrogation of mitochondrial ETC activity in *pdsw* ^i^ and *cova* ^i^ embryos. pAMPK was increased by 1.9 fold in *pdsw* ^i^ and 1.8 fold in *cova* ^i^ compared to control embryos (Fig. 5E,F). Levels of total AMPK-α in *pdsw* ^i^ and *cova* ^i^ embryos remained unchanged as compared to the controls (Fig. S3B,C). An increase in pAMPK was also observed in western blots from extracts obtained from *pdsw* ^i^ and *cova* ^i^ mutant embryos (Fig. 5G,H). There was no significant change in mitochondrial distribution and morphology across the apico basal axis in *pdsw* ^i^ and *cova* ^i^ expressing embryos (Fig. S3D,E).

We also tested whether administration of a glucose analogue 2-Deoxy Glucose (2DG) that inhibits glycolysis resulted in activation of pAMPK in the syncytial blastoderm embryo. We found that even though the concentration of 2DG was sufficient to increase pAMPK in the nucleus in follicle cells in oogenesis (data not shown), it did not cause any change in pAMPK levels in the syncytial blastoderm embryo (Fig. S4A,B) suggesting that glucose was not a major substrate for ATP production. This shows that glucose is not a major substrate for ATP generation in *Drosophila* embryo and these data are consistent with previous literature which shows that enzymes involved in the metabolism of glucose are predominant only at later stages of embryogenesis^51, 52^.

In summary, ATP production is reduced and pAMPK levels are elevated on acute as well as chronic inhibition of ETC. This shows that even though mitochondria are relatively small in the syncytial *Drosophila* embryo, their ETC is active.

### Inhibition of ETC decreases the metaphase furrow extension in syncytial *Drosophila* embryos

**Figure 6:**
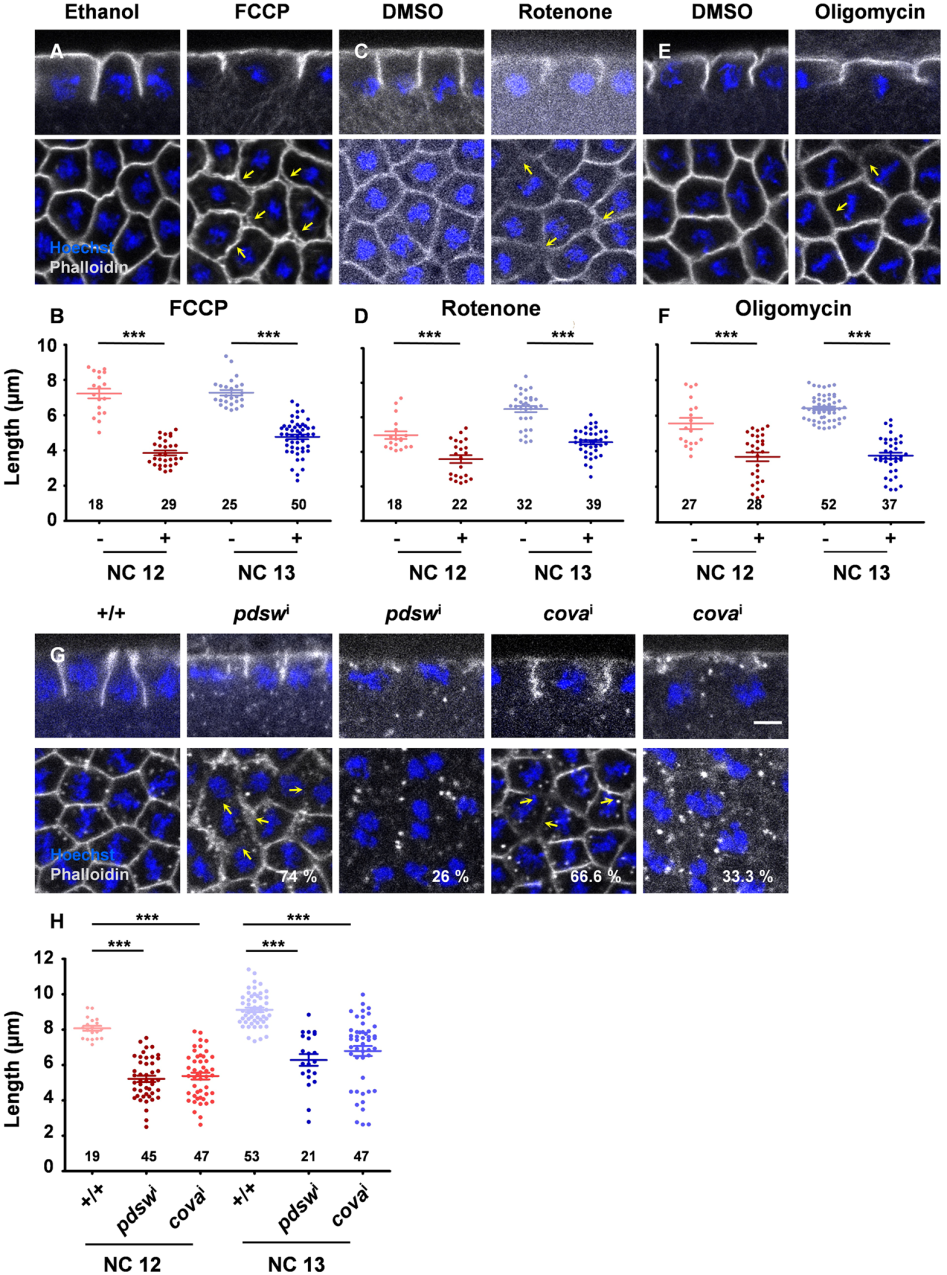
Inhibition of ETC decreases metaphase furrow extension in syncytial *Drosophila* embryos. (**A**–**F**) Pharmacological inhibition of ETC results in decrease in metaphase furrow length in NC12 and 13. Shorter metaphase furrows (Phalloidin; grey) are seen in FCCP compared to control in representative sagittal views through metaphase furrows and Surface view shows loose actin organisation (**A**). Furrow length similarly decreased in Rotenone (**C**) and Oligomycin (**E**) compared to respective controls. Quantification of metaphase furrow length shows a significant decrease (**B**,**D**,**F**) in NC12 and 13. Each data point represents individual metaphase furrow lengths, NC12 Control n = 18 (4 embryos), FCCP: 29 furrows (6); NC13 control n = 25 (6) and FCCP: 50 (11), (**B**); NC12 control n = 18 (5), Rotenone n = 22 (4); NC13 control n = 32 (5), Rotenone n = 39 (6) and (**D**); NC12 control n = 18 (4) and Oligomycin n = 28 (7) and NC13 control 52 (10) and Oligomycin 37 (7) (**F**) (***P ≤ 0.001, two tailed Mann Whitney test). Yellow arrows (**A**,**C**,**E**) mark loose membrane/actin on drug treatment. N: FCCP, Rotenone and Oligomycin-3. (**G**–**H**) Genetic inhibition of ETC results in decrease in metaphase furrow length in NC12 and 13. Metaphase furrows are shortened (74%) or completely absent (26%) in *pdsw* ^i^ compared to control (**G**). *cova* ^i^ metaphase furrows are short (66.6%) or absent (33.3%). Yellow arrows mark loosening or loss of the membrane/actin (**G**). Quantification of metaphase furrow lengths in *pdsw* ^i^ and *cova* ^i^ mutant as compared to control embryos (**H**) in NC12 and 13. NC12 control 19 (4 embryos) *pdsw* ^i^ n = 45 metaphase furrows (8) and *cova* ^i^ 47 (8), and; NC13 control 53 (8), *pdsw* ^i^ 21 (4) and *cova* ^i^ 47 (7) and (**H**) (***P ≤ 0.001, two tailed Mann Whitney test). N: *pdsw* ^i^-4, *cova* ^i^-3. Scale bar: 5 µm.

We further explored the functional consequence of abolition of mitochondrial ETC activity on the syncytial division cycles. Syncytial nuclei are covered partially on the apical side by the plasma membrane. During metaphase of syncytial divisions, the plasma membrane extends basally in between adjacent spindles, forming metaphase furrows^38, 53^. This process is driven by rapid actin assembly and could be dependent on a continuous supply of ATP. Therefore we tested whether ATP depletion using acute treatment of ETC inhibitors affects metaphase furrow extension. Syncytial embryos treated with FCCP, Rotenone and Oligomycin were analysed for metaphase furrow lengths using fluorescently labelled Phalloidin, which marks F-actin. We found a significant decrease in metaphase furrow lengths in both NC12 and 13 in all these treatments (Fig. 6A–F). Embryos expressing *pdsw* ^i^ and *cova* ^i^ also exhibited a furrow shortening and membrane loosening phenotype (Fig. 6G,H). In addition, 26% of *pdsw* ^i^ and 33% of *cova* ^i^ embryos showed absence of Phalloidin staining indicating a complete loss of metaphase furrow formation (Fig. 6N,L). We did not find differences in metaphase furrow length of NC12 and 13 2DG treated embryos compared to controls (Fig. S4C,D).

We also tested if change in mitochondrial distribution in *khc* ^i^ embryos affected pAMPK levels and found that there was no significant difference in immunostaining of pAMPK (Fig. S4A,B). This is likely because mitochondria are abundant in the syncytial blastoderm embryo and a change in the mitochondrial distribution will not substantially activate AMPK to increase pAMPK. The metaphase furrow length was decreased in *khc* ^i^ embryos in NC13 (Fig. S4C,D) and this is in agreement with the previous observations and is because of reasons unrelated to change in mitochondrial density and activity^54, 55^.

In summary, our study shows that mitochondria in the syncytial *Drosophila* embryo are localized in proximity to microtubules and do not move to neighbouring syncytial cells. This local mitochondrial distribution is important for producing ATP from ETC activity to support the syncytial division cycles, especially for metaphase furrow formation.

## Discussion

We have characterized mitochondrial distribution and dynamics in syncytial *Drosophila* embryos and have found that ATP supply from the ETC in relatively small and dispersed mitochondria is essential for metaphase furrow formation during the syncytial division cycles. We discuss the implications of our data in three distinct parts concentrating on mitochondrial distribution in the context of the syncytial architecture, the role of cytoskeleton-mediated transport for maintaining this distribution and the function of the mitochondrial ETC in early blastoderm divisions.

**Figure 7:**
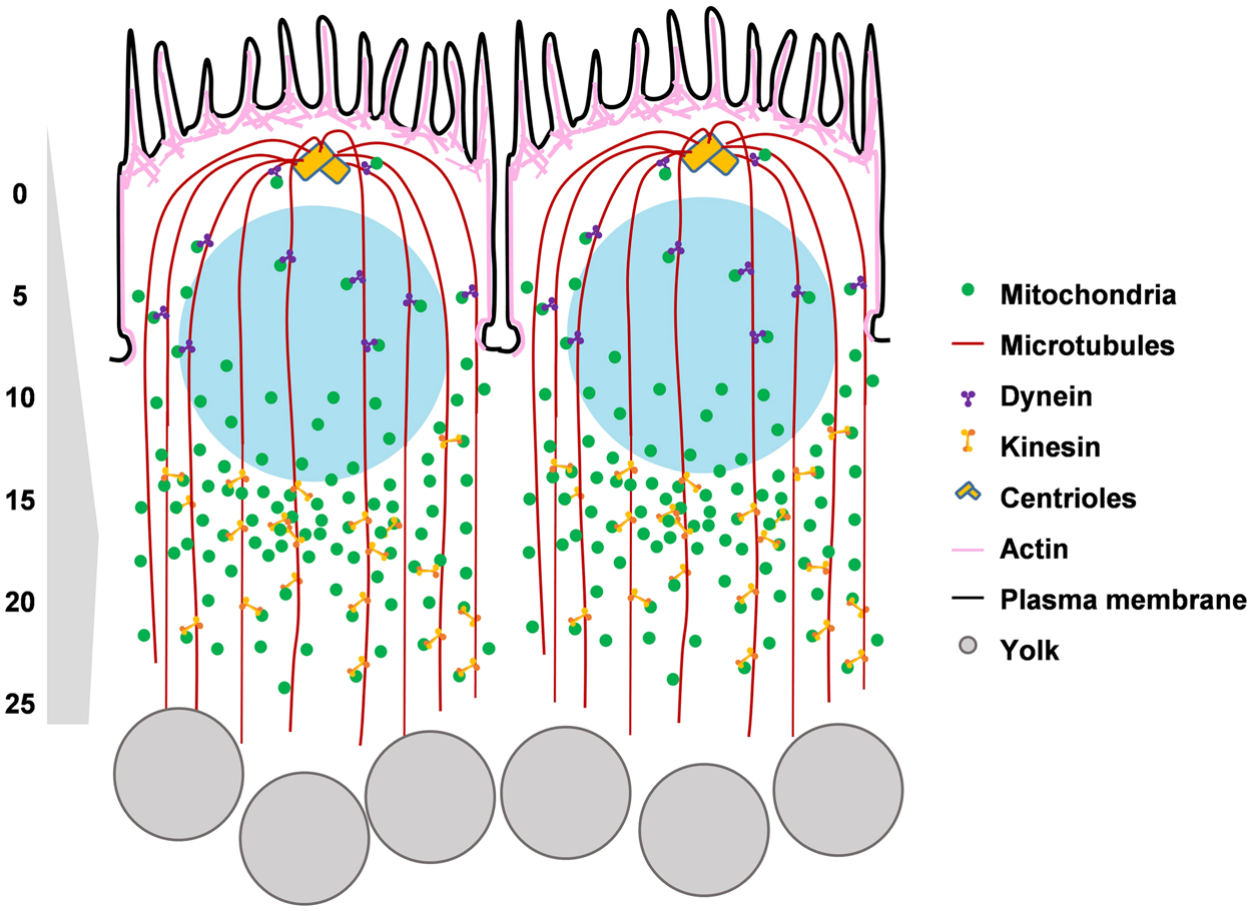
Summary of mitochondrial distribution and function in the *Drosophila* syncytial cycles. The syncytial *Drosophila* embryo contains dispersed mitochondria, which increase in density towards basal region of syncytial cells. Mitochondrial movement is restricted within one syncytial cell. Apico-basal concentration of mitochondria is dependent on an intact tubulin network in the syncytial blastoderm embryo. Mitochondrial ETC activity is essential for ATP production in the syncytial cells for the process of metaphase furrow formation during syncytial cycles of the *Drosophila* embryo.

Contrasting the maternally inherited ER and Golgi complex, mitochondria are present around preblastoderm nuclei located deep in the embryo. Centrosomes and spindles mediate nuclear divisions^38^ in the interior of the embryo and mitochondria are likely to be important as a local energy source for these nuclear cycles. Despite absence of complete plasma membrane boundaries, mitochondria are restricted to individual syncytial cells and not shared between neighbouring cells (Fig. 7) in accordance with previously studied ER and Golgi complexes^23^ in the syncytial blastoderm embryo. Compartmentalization of organelles and cytoplasm is important for confinement of biological activities of protein synthesis and delivery close to individual nuclei in syncytial cells of different origins. The distribution of mitochondria with nuclei in the interior of the *Drosophila* embryo along with their discrete distribution around the syncytial nuclei argues for their function in providing ATP in the vicinity of energy intensive, morphogenetic processes of furrow formation. Multinucleate muscle cells and fungi show restricted spread of transcription and translation products around one nucleo-cytoplasmic domain and this is important for eliciting local regulation of cell division and signalling^56–60^. Our data show that mitochondrial ETC activity is essential for membrane ingression in embryonic syncytial division cycles and hence presence of mitochondria in the vicinity allows abundant and local support for energy demands of the syncytial cycle. The restricted mitochondrial distribution also ascertains even distribution of mitochondria from mother to daughter syncytial cells in the proliferating embryo. Proper segregation of mitochondria in yeast daughter cells is essential for their survival^61^.

Microtubules are predominantly apico-basally oriented in interphase of syncytial cells and form a cage around nuclei, with centrosomes present above and plus ends of microtubules extending below the nuclei^38^. We found basal enrichment of mitochondria along with significant apico-basal movement and negligible planar movement (Fig. 7). These data along with the observed localization of mitochondria in the proximity of microtubules are consistent with the explanation of mitochondria being stably associated with microtubules in the syncytial embryo. Microtubules traffic Golgi complexes to the apical direction during late phase of *Drosophila* cellularization^30^. Mitochondria bind to microtubule motors Kinesin and Dynein with linker proteins Miro, Milton and Dynactin^18, 19, 21, 36, 62, 63^. Mitochondrial biogenesis occurs in cell bodies of neurons^64^ and locally in the axons^65^. They are transported via Kinesin and docked at axonal termini^66–68^ via growth signaling^69, 70^. Retrograde transport via Dynein motors enables recycling of damaged mitochondria^36, 71^. Mitochondrial interaction with Klp67A has been shown before in *Drosophila* syncytial embryos^72^, but microtubule association and regulation of mitochondrial trafficking in syncytial cells is not clear. We found that mitochondria were depleted in the apico-lateral region in syncytial cells when microtubules were depolymerized. Reduction of plus end directed Kinesin motors resulted in mitochondrial clustering in the apico-lateral region, indicating that this transport maintains mitochondria basally (Fig. 7). The apical increase in number of mitochondria during metaphase, anaphase and telophase could be regulated by minus end motors. Apical transport of mitochondria has not been tested in our study and further studies on mitochondrial trafficking in the apico-basal axis by motors will help to clarify their differential activity in maintaining appropriate concentration at specific cellular regions in the syncytial *Drosophila* embryo.

The syncytial *Drosophila* embryo contains small and dispersed mitochondria which are correlated in literature with poor ATP generation^40, 41^. *Drosophila* embryos contain an abundance of maternally deposited substrates that may be broken down to generate ATP. Despite a lipid droplet rich yolk, the 0–2 hr old embryo mainly relies on amino acid metabolism from aspartate and glutamate which provide substrates for operating the ETC^42^. Hence ETC is the major mode of ATP synthesis in early embryo. This is consistent with previous observations in ascidian and mammalian embryos where ATP is generated by the ETC and the ATP status is also linked with calcium homeostasis^7, 9^. Genetic and pharmacological ETC depletion in our studies increased pAMPK. pAMPK levels remained unchanged on administration of 2DG, thus confirming that metabolites of glycolysis are not the major substrates for the ETC in *Drosophila* syncytial blastoderm embryos. AMPK phosphorylation by LKB-1 serves as a biological marker for lowered ATP^73–75^. AMPK activation during stress in mammalian cells leads to its localization into the nucleus where it binds and phosphorylates FOXO to induce transcription to maintain energy balance by reducing catabolism in the cell^76^. pAMPK is predominantly present in syncytial nuclei and potentially affects transcription regulation^77^. Its further elevation in embryos treated with pharmacological and genetic inhibitors of ETC confirms effectiveness of these treatments and an operation of this pathway for meeting energy demands in the syncytial embryo. Mitochondrial ETC inhibitors lead to mitochondrial fragmentation^78^. However, we did not observe any change in mitochondrial morphology or distribution with these treatments as compared to controls. Future experiments that significantly deplete mitochondria in the apico-basal axis in the syncytial blastoderm will be needed to test the direct link between mitochondrial activity and local requirement.

pAMPK regulates cell shape via modulation of Myosin regulatory light chain^79^ and AMPK mutants show increased filamentous to globular (F/G) actin ratio in adult *Drosophila* brains^80^. It is possible that increased pAMPK in syncytial embryos leads to decrease in F/G ratio and this imbalance consequently results in shorter metaphase furrows. Metaphase furrow formation in syncytial *Drosophila* embryos is regulated by actin regulator RhoGEF^81^ and Dynamin^82^ and lowered ATP could impact the activity of these proteins^83, 84^ thereby resulting in shorter furrows. ATP in the cells is reduced in hypoxic conditions since oxygen is required for metabolism of substrates by ETC. Oxygen deprivation causes cell cycle arrest^85^ and impairs mitotic spindles^86^ in *Drosophila* embryogenesis. We have not tested whether depletion of mitochondrial ATP and increased pAMPK affects metaphase spindle assembly and may be additionally responsible for shorter furrows.

In summary, we have attempted to initiate early *Drosophila* embryogenesis as a model to study the role of mitochondrial dynamics and distribution during development. Mitochondria are enriched at the blastopore lip during gastrulation in *Xenopus* embryos^87^. Mitochondria localize to prospective oral axis of Sea urchin embryos and dysregulation of this distribution perturbs axis specification^88, 89^. These studies allow a speculation on the possibility of specific regulation of mitochondrial distribution and activity during embryogenesis. Mitochondria may regulate calcium, reactive oxygen species and signaling in addition to ATP generation in the embryo. Future studies on mitochondrial localization and function in the early embryo will reveal how their different functions might interact with embryogenesis via developmental cues that regulate cellular processes in syncytium, cellularization and gastrulation.

### Methods

#### Fly Stocks

*Drosophila* crosses were maintained at 25 °C in standard cornmeal agar. *khc* ^i^ crosses were maintained at 29 °C. *nanos*-Gal4 was used to induce the expression of fluorescently tagged transgenes and RNAi against Pdsw, Cova and Khc in oogenesis. *nanos*-Gal4, Mito-GFP recombinant line was made by standard genetic crosses. UASp-Mito-GFP was obtained from Rachel Cox. *nanos*-Gal4, *pdsw* ^i^, *cova* ^i^, *khc* ^i^, H2A-RFP, Tubulin-mCherry and Moesin-mCherry were obtained from Bloomington stock center. UASp-KDEL-RFP, UASp-GalT-RFP and UASp-Mito-PAGFP were made as described below.

### Cloning

#### pUASP-KDEL-RFP

The primers ATTTGCGGCCGCATGGACAGCAAAGGTTCGTC and GCTCTAGAGGATCCTTAGAGCTCATCTTT containing the Not1 and Xba1 restriction enzyme sites respectively were used to amplify the lyso-RFP-KDEL sequence, restriction digested and cloned into the pUASP vector. lyso-RFP-KDEL was constructed by fusing the complete coding sequence of hen’s egg lysozyme with the COOH terminal RFP followed by a KDEL ER retention signal^90^. Embryo injections and transgenic animal selection was carried out at the Best Gene Transgenic Facility, CA, USA.

#### pUASP-GalT-RFP

The primers ATTTGCGGCCGCATGAGGCTTCGGGAGCCG and GCTCTAGATTAGGCGCCGGTGGAGTG containing the Not1 and Xba1 restriction enzyme sites respectively were used to amplify the GalT-RFP sequence, restriction digested and cloned into the pUASP vector. The GalT-RFP sequence contains 1–60 amino acids of the human galactosyltransferase sequence including the amino terminus cytoplasmic tail, uncleaved signal sequence and 17 amino acids of the lumenal domain fused to full length RFP (The construct was gifted by Jennifer Lippincott-Schwartz, NIH). Embryo injections and transgenic animal selection was carried out at the Best Gene Transgenic Facility, CA, USA.

#### pUASP-Mito-PAGFP

The Mito-YFP construct in the pUASP vector was obtained from Rachel Cox. As described in Cox and Spradling 2003, this construct contains the mitochondrial targeting sequence from human cytochrome oxidase VIII fused to YFP. The PAGFP-N1 vector was restriction digested with BamH1 and Xba1 and the PAGFP fragment obtained was subcloned into the pUASP-Mito-YFP vector to replace YFP with PAGFP. The pUASP-Mito-PAGFP vector injection into *Drosophila* embryos and selection of transgenic animals was done by the NCBS injection facility, Bangalore, India.

#### Immunohistochemistry

F1 flies containing *nanos*-Gal4 and fluorescently tagged transgene or RNAi were selected from crosses and transferred to embryo collection cages (Genesee Scientific, CA, USA) with 3% sucrose agar supplemented with yeast paste. Embryos were washed, dechorionated using 100% bleach for 1 min, washed and fixed in heptane: 4% paraformaldehyde (1:1) in PBS (137 mM NaCl, 2.7 mM KCl, 10 mM Na_2_HPO_4_, 1.8 mM KH_2_PO_4_) for 20 mins at room temperature. Embryos were devitellinized by vigorously shaking in heptane:methanol (1:1) for α-tubulin, pAMPK and total AMPK staining or hand devitellinized for Phalloidin staining. Blocking was done using 2% BSA (Bovine Serum Albumin) in PBS with 0.3% Triton X-100 (PBST). Following primary antibodies were diluted in the Block solution: pAMPK 1:200 (Cell Signaling, MA, USA), AMPK-α 1:200 (Abcam, MA, USA), α-Tubulin 1:500 (Sigma-Aldrich, Bangalore, India). Fluorescently coupled secondary antibodies (Alexa Fluor 488, 568, 633, Molecular Probes, Bangalore, India) were used at 1:1000 dilution in PBST. Fluorescently tagged Phalloidin (1:500, Molecular Probes, Bangalore, India) and Streptavidin (1:1000, Molecular Probes, Bangalore, India) were added with secondary antibodies. DNA was labelled using Hoechst 33342 (1:1000, Molecular Probes, Bangalore, India). Embryos were imaged using Plan apochromat 40X/63X/1.4 objectives on the Zeiss LSM710/780.

#### ETC disruption, glycolysis inhibition and Tubulin depolymerization drug treatment

Embryos of desired stage were dechorionated in 100% bleach, permeabilized with D-Limonene (Sigma):Heptane (1:1) (LH) containing drugs and incubated at RT for the mentioned time^49^. Embryos were then fixed and stained as mentioned above. Drug concentrations and incubation times used were, FCCP: 10 µM (Sigma Aldrich, Bangalore, India); 15 mins, Rotenone: 5 µM; 30 mins (Sigma Aldrich, Bangalore, India), Oligomycin: 10 µM; 5 mins (Sigma Aldrich, Bangalore, India), 2-Deoxy-D-glucose: 100uM; 15 mins (Sigma Aldrich) Nocodazole: 330 nM; 20 mins (Sigma Aldrich, Bangalore, India). 10 mM FCCP stock was prepared in ethanol and 5 mM Rotenone, 10 mM Oligomycin, 3.3 mM Nocodazole and 5 mM 2-DG were made in DMSO. Treatment of control embryos with an equivalent concentration of ethanol and DMSO was done at the same time. 2DG was tested in *Drosophila* follicle cells during oogenesis for AMPK activation and pAMPK was found in the nucleus as compared to controls (data not shown). 2DG was added along with 5 µM FM464-FX (Molecular Probes) in order to probe permeabilization of embryos for entry of 2DG.

#### Live Imaging

1.5 hour old embryos were collected from sucrose agar plates, washed, dechorionated, mounted on coverglass chambers (LabTek, Germany) in PBS and imaged using Plan Apochromat 40x/1.4 objective on a Zeiss LSM780 at an optical zoom of 3x. Consequent Z stacks with 30 images of 1 µm optical thickness were taken with a scan speed of 1 sec per image. Samples were imaged using 488 nm and 561 nm lasers at 2% power with an appropriate gain, within the dynamic range for imaging GFP and RFP or mCherry respectively.

#### Photobleaching

Continuous photobleaching of the region of interest (ROI) of size 4 μm^2^ was carried out in syncytial embryos expressing Mito-GFP with 488 nm laser at 100% power and 30 iterations. The ROI was bleached after every 18 seconds. The same and neighbouring syncytial cell was monitored for fluorescence depletion. Images were acquired with 488 nm laser at 2% using Plan Apochromat 40x/1.4 NA objective on a Zeiss LSM780 at 3x zoom.

#### Photoactivation

Photoactivation on syncytial embryos expressing Mito-PAGFP was performed in selected ROIs (either complete or half syncytial cell) using the 405 nm laser with 100% power and 30 iterations by Plan Apochromat 40x/1.4 NA objective on the Zeiss LSM780. Images were acquired using 488 nm laser at 2% power. For calculation of relative intensities of daughter syncytial cells in nuclear divisions, the ROI covering the entire syncytial cell was activated.

#### ATP estimation

ATP estimation was carried out from embryo extracts by using luciferase based ATP determination Kit (Thermofischer scientific). Briefly, 3 hr old embryos were collected and rinsed in heptane twice and subsequently dried completely. Embryos were manually crushed on ice using 1.5 ml microfuge tube pestle in homogenisation buffer (Tris (100 mM) and EDTA (100 µM)) till a uniform extract was obtained. The extract was lysed by boiling for 5 min and the supernatant was collected by spinning at 21000 g at 4 °C. Supernatant was diluted (1:10) in dilution buffer (25 mM Tris, 100 µM EDTA) and again spun at 21000 g at 4 °C. After diluting appropriately, Luciferin and firefly luciferase in buffer provided in the kit were added to the samples in 96 well white plates and ATP concentration dependent luminescence was measured immediately on a Varioskan Spectrometer at 560 nm. In order to ascertain reproducibility, both experimental and control samples were assayed at three different dilutions. Each dilution was loaded in triplicates and readings for the entire plate were taken thrice. All measurements were normalized to total protein content of the embryos. Protein estimation was done using BCA kit (Thermofischer scientific) against standard BSA concentrations. Each sample was loaded in three different wells and emission for each well was measured thrice. All the experiments were repeated 3 times. The graph represents the percentage reduction corresponding to controls estimated at the same time. Means were compared using one tailed Mann-Whitney test in Graphpad prism 6.0.

#### Western Blotting

WT and mutant embryos aged 2.5 hrs were collected and dechorionated using 100% bleach for 1 min. They were homogenized in a centrifuge tube using 150 µl of lysis buffer (1%Triton-X 100, 50 mM Tris HCl; pH 8.0, 150 mM NaCl, Protease Inhibitor Cocktail; PIC 1:50). The homogenate was sonicated for 50 sec with successive on and off cycles of 5 seconds each followed by centrifugation at 14000 g at 4 °C for 10 mins. Supernatant was stored at −80 °C and protein concentration was estimated using BCA kit (Thermo Fisher Scientific). After addition of gel loading buffer and heating at 95 °C for 10 mins, equal protein concentration (15 µg) was loaded in 10% SDS gel wells and separated at 90 V. Separated proteins were blotted onto PVDF membrane, activated with methanol, at 4 °C at 90 V for 3 hrs. Blot was washed with TBST and blocked using 5% milk for 1hr at room temperature and then incubated with rabbit anti pAMPK antibody (1:1000, Cell Signaling), mouse anti AMPK (1:1000, Abcam) and mouse anti beta-tubulin (1:10,000, Sigma) overnight at 4 °C followed by 3 washes with 1X TBST. Blot was then incubated with respective HRP conjugated secondary antibodies for 1hr at room temperature and developed using ECL prime blot detecting reagent in ImageQuant™ LAS 4000. Blots probed with pAMPK were cut, stripped and reprobed with AMPK-α and Tubulin to remove brighter non-specific bands. pAMPK and total AMPK signal of control and mutant samples was estimated using densitometric quantification of band intensities across independent blots in Image J. pAMPK intensity was normalized to the corresponding total AMPK intensity in each sample and fold change was calculated with respect to control and plotted. Mean values obtained from 3 experiments were compared using unpaired Student’s t-test in Graphpad Prism 6.0 software.

### Image analysis

#### Mitochondrial fluorescence across syncytial cycle

Mean mitochondrial fluorescence intensities were measured in optical sections from NC10 to 13 interphase, corrected using minimum intensity value and normalized to maximum intensity for individual NCs. Average of 3 embryos was plotted against depth with standard error.

#### Mitochondrial object number and density measurement

Discrete mitochondrial objects visualized as punctae in apical sections were intensity thresholded and total numbers of optically separable objects in a fixed apical optical plane were measured using particle analyser tool with a threshold cut-off of 0.05 μm^2^ in ImageJ for every cell cycle stage of NC12. Mitochondrial punctae per syncytial cell in NC12 were compared using one-way Kruskal Wallis test. Total number of punctae in 12I and 13I were compared using two-tailed Mann-Whitney test. Mitochondrial density was measured by obtaining total mitochondrial area post thresholding and normalization with total imaging field area in nocodazole treated embryos and *khc* ^i^ embryos and compared with corresponding controls using two tailed Mann-Whitney test using Graphpad prism 6.0 software.

#### FLIP and Photoactivation

Average fluorescence intensities of ROIs of different time series were measured using Time Series Analyser plugin in ImageJ and background corrected using minimum intensity value of entire imaging field for each image. Intensities of the whole time series were normalized to pre-bleach intensity and post activation intensity values for FLIP and photoactivation respectively. Mean of 3 embryos was plotted with standard error. To calculate relative intensities in daughter cells after photoactivation, ROIs were drawn around photoactivated cells and their daughter cells and mean fluorescence intensities of ROIs were measured using ImageJ and corrected using average background intensity for each in interphase of NC11, 12 and 13 respectively. Average fluorescent intensities of all NC12 and 13 daughter cells of photoactivated NC11 mother cell in each embryo were calculated and normalized to mean fluorescence intensity of NC11 mother cells in individual embryos. Histogram represents average of normalized intensities across 5 embryos. Means were compared using Student’s t-test using Graphpad prism 6.0 software.

#### pAMPK

Wild type control embryos were collected on the same day and divided into two aliquots for treatment with the solvent alone or the drug. Likewise *pdsw* ^i^ and *cova* ^i^ embryos were collected along with control embryos into different vials. The primary and secondary antibody dilutions were made in bulk and used in corresponding vials. The corresponding control/treated or control/RNAi embryos were imaged at the same time keeping the laser power and gain constant. The sample, which was stained more intensely, was used to adjust the laser power and the gain and the corresponding control was imaged at the exact same settings. Mean fluorescence intensity was measured across the whole optical plane and the minimum fluorescent intensity value was subtracted from each image. An average intensity was obtained from the respective control images and the treated/mutant/control fluorescence was normalized to this value to obtain a fold change in intensity across samples. Distributions were compared using two-tailed Mann- Whitney test on Graphpad prism 6.0 software.

#### Metaphase furrow length

Lines were drawn along metaphase furrows in sagittal section images and lengths were measured in ImageJ for NC12 and 13. Mean furrow lengths were compared using two tailed Mann-Whitney test on Graphpad prism 6.0 software.

#### Data Availability

The datasets generated during and/or analysed during the current study are available from the corresponding author on reasonable request.

## Electronic supplementary material

Supplementary legends and figures

Movie S1

Movie S2

Movie S3

Movie S4
