# Supplementary material for: Analysis of mitochondrial organization and function in the Drosophila blastoderm embryo
Source: Sci Rep. 2017 Jul 14;7:5502. doi: 10.1038/s41598-017-05679-1 (PMC5511145; doi:10.1038/s41598-017-05679-1)
Supplement: Supplementary file 1 — Supplementary legends and figures [file 41598_2017_5679_MOESM1_ESM.pdf]

Analysis of mitochondrial organization and function in the *Drosophila* blastoderm embryo

Sayali Chowdhary, Darshika Tomer, Dnyanesh Dubal, Devashree Sambre and Richa Rikhy*

Affiliation and contact information:

Biology, Indian Institute of Science Education and Research, Homi Bhabha Road, Pashan, Pune, 411008, India

Phone: +91-20-25908065

* To whom correspondence is addressed: richa@iiserpune.ac.in

Supplementary Figures

Figure S1: Mitochondrial distribution with respect to ER, Golgi and actin in the syncytial *Drosophila* embryo

A: Mitochondrial distribution with respect to ER. Embryos expressing Mito-GFP (green) and KDEL-RFP (red) are imaged live and optical stacks across depth are shown in NC13.

B: Mitochondrial distribution with respect to Golgi. Embryos expressing Mito-GFP and Galt-RFP (red) are imaged live and optical stacks across depth are shown in NC13. Galt-RFP is also present as punctate structures and increase in concentration across the depth of the embryo (representative images from 3 movies for A, B).

Scale: 5 µm

Figure S2: Partial mitochondrial photoactivation in syncytial cells shows asymmetric distribution of photoactivated mitochondria in the daughter cells formed on that side.

A: Streptavidin colocalizes with Mito-PAGFP. Mito-PAGFP (green) containing NC 13 embryos are stained with fluorescent conjugated Streptavidin (red).

B-C: Photoactivated mitochondria on one side of mother syncytial cell (light red) are distributed to the daughter cell formed on the same side (red, 2) and not to the other side (green, 1). The intensity is shown as a pseudocolored rainbow scaled image. Normalized fluorescence intensity is shown in the two daughter cells (C). N = 4, (***, $P \leq 0.001$, two tailed Student's t test).

Figure S3: Total AMPK levels do not change on genetic depletion of ETC

A: pAMPK signal is cytoplasmic and nuclear in control embryos based on the cell cycle. Nuclear signal of pAMPK is enhanced in prophase embryos and is more on kinetochore in metaphase. The pAMPK fluorescence is enhanced for visualization as compared to control images in Fig. 5.

B-C: Total AMPK-α is seen more uniformly distributed in the control syncytial embryo, pAMPK (green in merge) is increased significantly in $pdsw^i$ and $cova^i$ expressing embryos and seen in the nucleus and at the centromere, however total AMPK-α (red in merge) intensity is not changed.

D-E: Mitochondrial density at apico-lateral regions in not affected in $pdsw^i$ and $cova^i$ compared to control in optical sections shown in D and quantified in graph shown in E (n = control 6, $pdsw^i$ 6, $cova^i$ 5, N=3). The control data for mitochondrial density is the same as Fig 4I-J.

Scale: 5 µm

F: Representative full length blots of Fig. 5G represent pAMPK, AMPK-α and tubulin bands in WT, *pdsw*^{i} and *cova*^{i} embryos. Part of the pAMPK blot was stripped and reprobed with AMPK-α followed by Tubulin. pAMPK levels are higher in *pdsw*^{i} and *cova*^{i} as compared to WT (1st blot); whereas AMPK-α (2nd blot) and Tubulin (3rd blot) levels are comparable.

Figure S4: Inhibition of glycolysis by 2-DG treatment does not alter pAMPK levels and metaphase furrow length.

A-B: Treatment of syncytial *Drosophila* embryos with 2-DG does not show increase in the levels of pAMPK (green-merged). n = 13, 13 embryos and 310 and 300 cells, N=2; for treated and control embryos respectively. (ns, P>0.05, two tailed Mann Whitney test).

C-D: Metaphase furrow length is not reduced in 2-DG treated embryos compared to control embryos. NC 12 control metaphase furrows n = 30 furrows (8 embryos), 2DG n = 17 (5); NC13 control n = 32 (7), 2DG n = 17 (5), N=3. (ns, P>0.05, two tailed Mann Whitney test)..

Figure S5: Kinesin depletion shows decrease in the metaphase furrow length but no activation of AMPK

A-B: Depletion of Khc does not show significant difference in pAMPK levels (red merged, A). n = 15, 16 embryos and 350, 400 cells; for *khc*^{i} and WT respectively (B) (ns, P>0.05, two tailed Mann Whitney test). N=3

C-D: Metaphase furrow length is decreased in NC13 *khc*^{i} expressing embryos compared to control. The control embryos are the same as Fig.6G-H. n = 15 (4 embryos), 53 (8 embryos) furrows for *khc*^{i} and WT respectively (B) (***, P ≤ 0.001, two tailed Mann Whitney test). N=3

Supplementary Movies

Movie S1: Mitochondria show restricted lateral movement in the syncytial embryo. Mitochondria in Mito-PAGFP expressing embryos are photoactivated in one syncytial cell (red), the fluorescence represented by a pseudocolored rainbow is restricted to the same cell (red) and the fluorescence does not change in the neighboring cell (green). Time is shown in min. Scale Bar is 10 µm.

Movie S2: Mitochondria are distributed equally from mother to daughter cells in the syncytial embryo. Mitochondria in Mito-PAGFP expressing embryos are photoactivated in one syncytial cell (red), the fluorescence represented by a pseudocolored rainbow distributes to the daughter syncytial cells (yellow and cyan). Time is shown in min. Scale Bar is 10 µm.

Movie S3: Mitochondria-GFP and Histone-RFP in apical caps in NC12. The movie shows a time lapse video of an embryo expressing Mito-GFP and Histone2A-RFP in NC12. The middle image is an apical section above the nucleus with orthogonal planes in the top and right image. Time is shown in min. Scale Bar is 10 µm.

Movie S4: Mitochondria-GFP and Histone-RFP in apico-lateral regions in NC12. The movie shows a time lapse video of an embryo expressing Mito-GFP and Histone2A-RFP in NC12. The middle image is an apico-lateral section through the nucleus with orthogonal planes in the top and right image. Time is shown in min. Scale Bar is 10 µm.

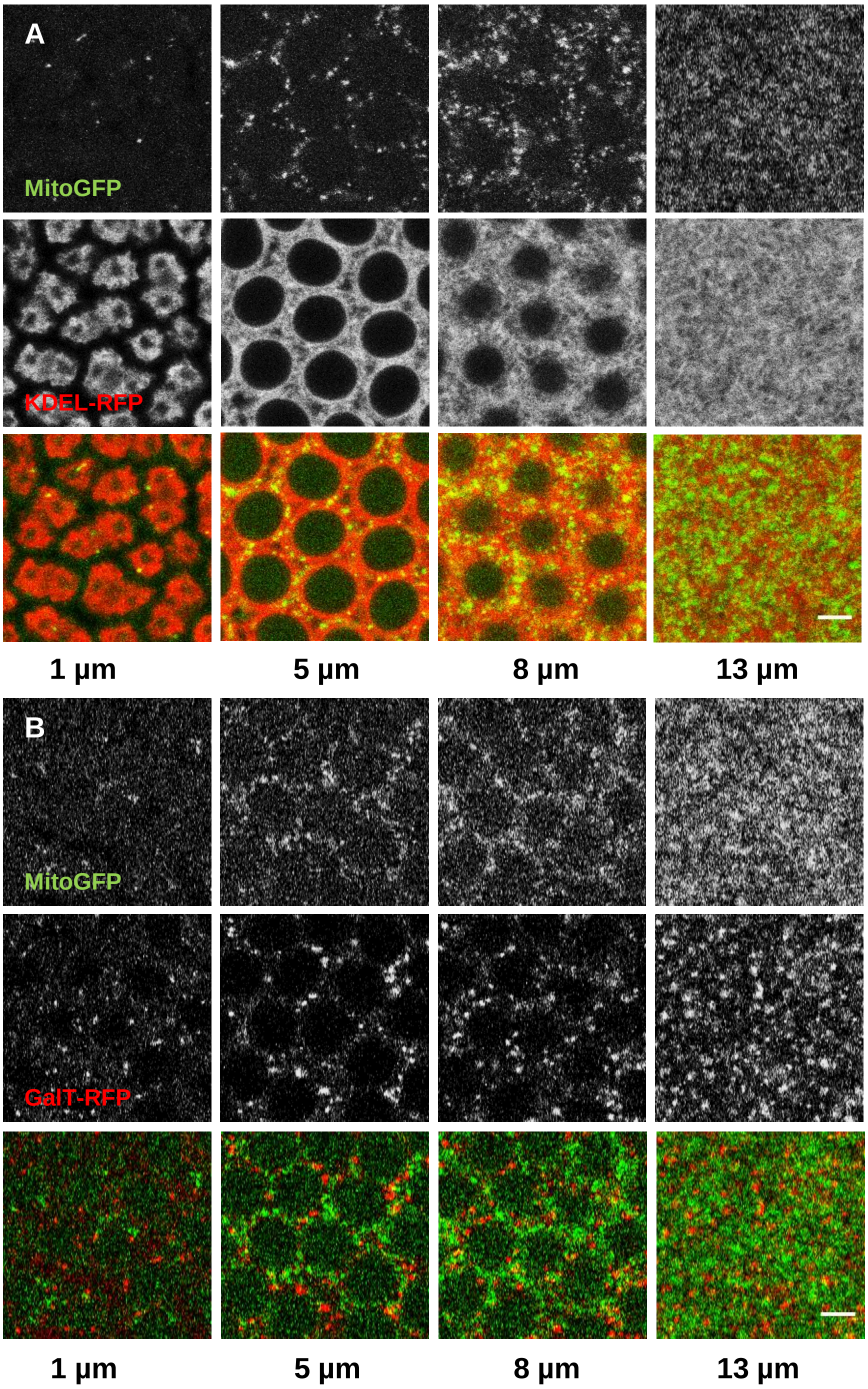

Chowdhary et. al. Figure S1

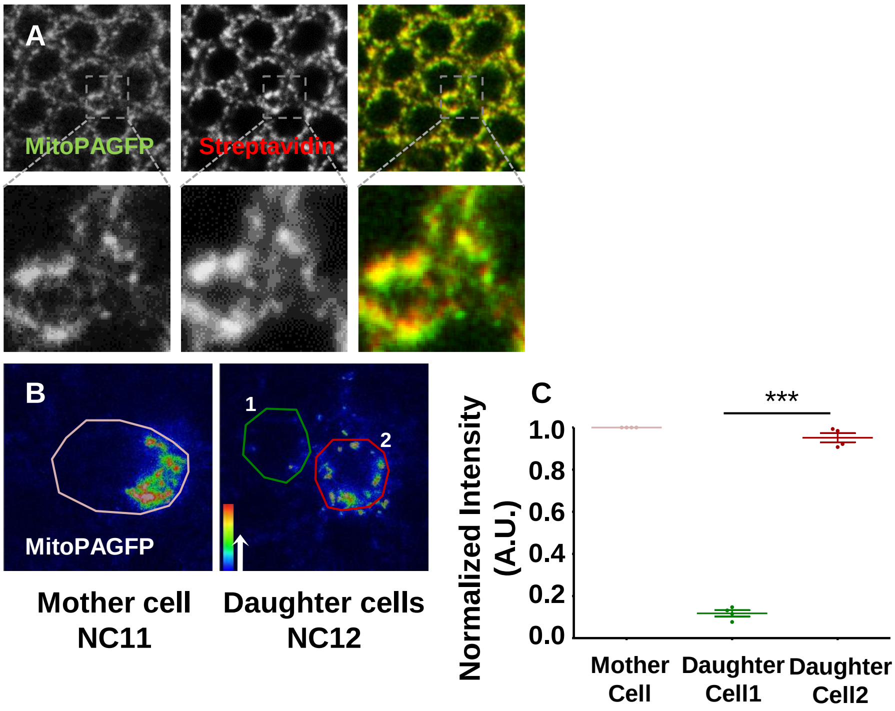

Chowdhary et. al. Figure S2

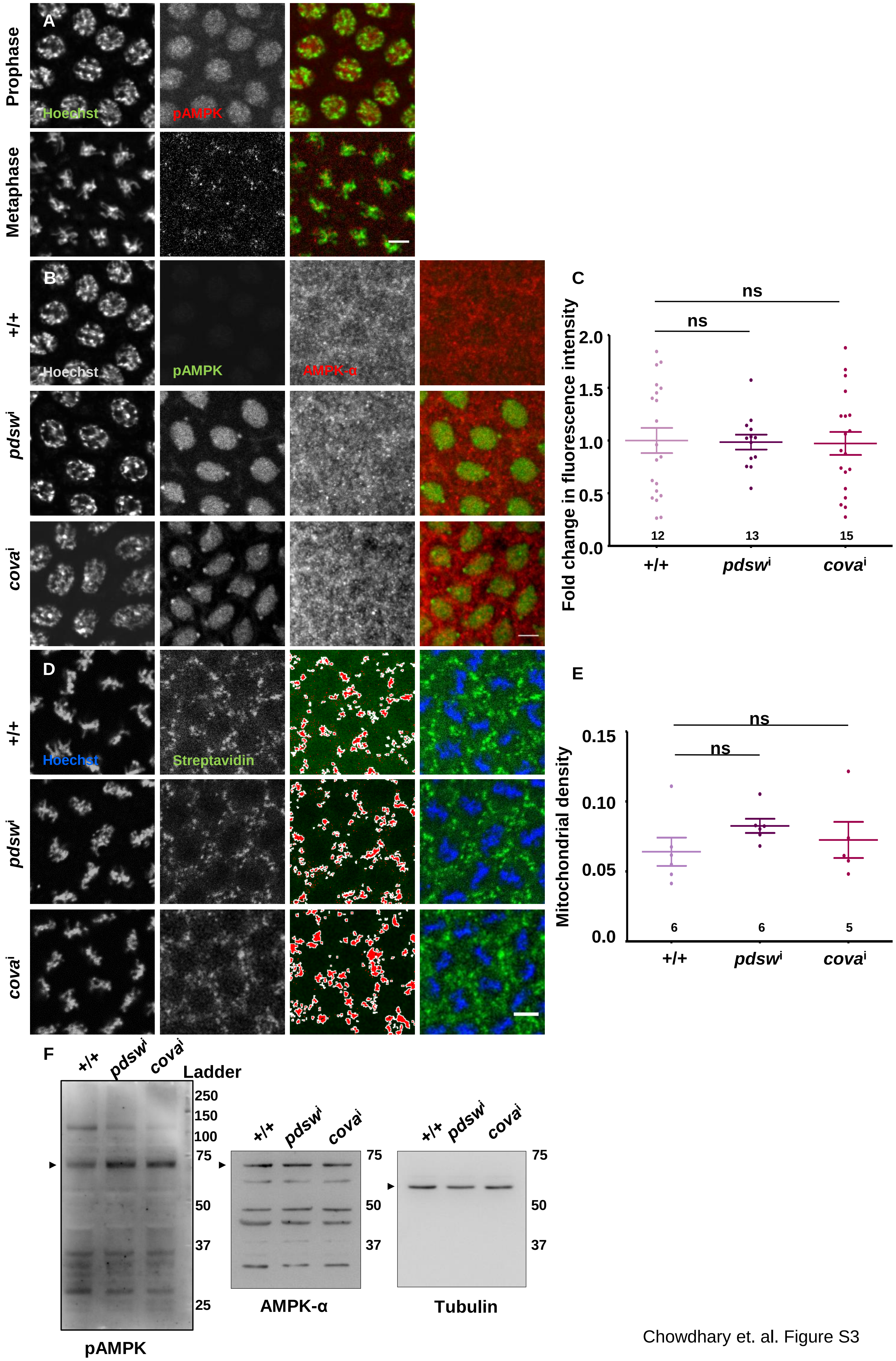

Chowdhary et. al. Figure S3

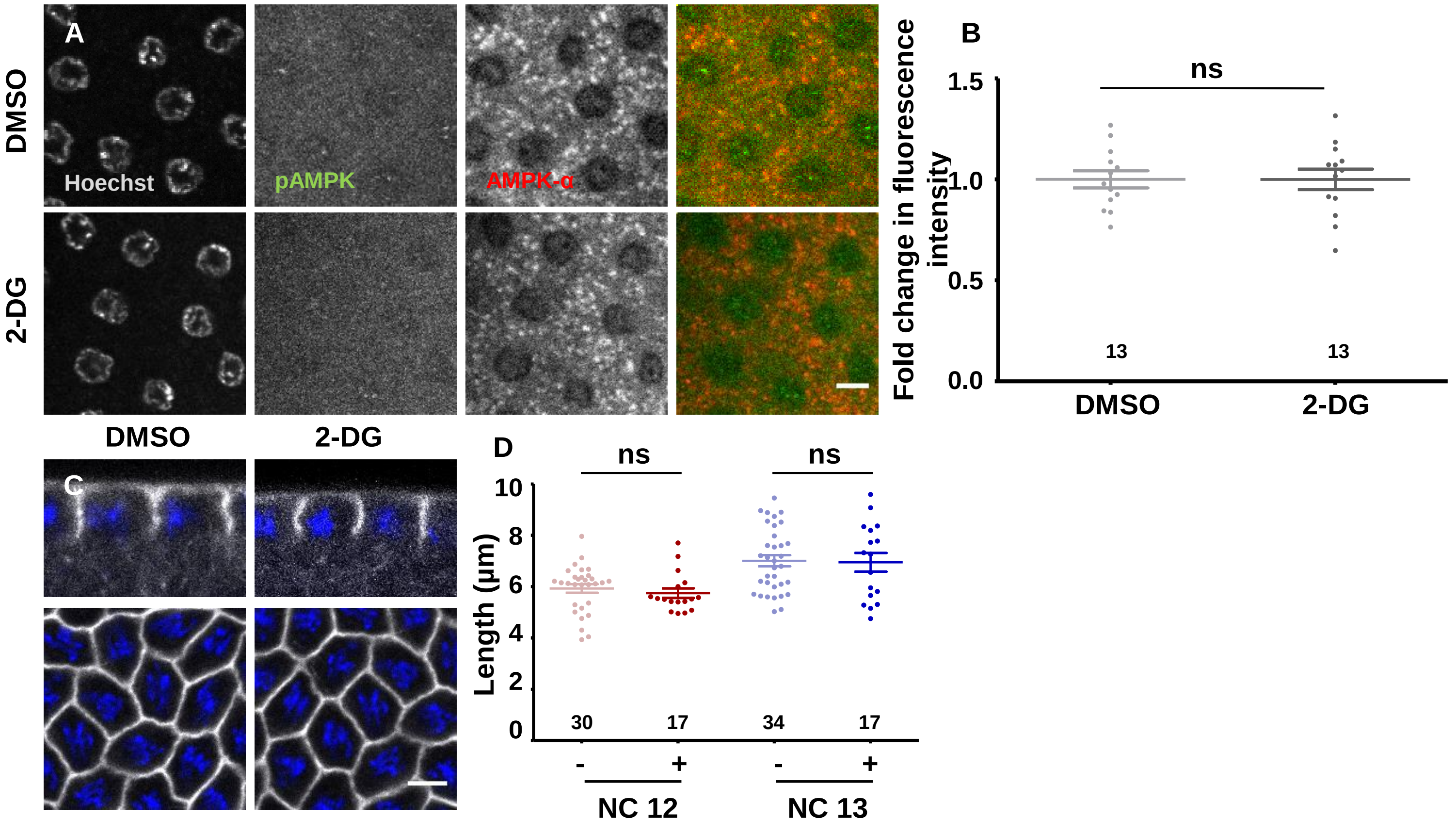

Chowdhary et. al. Figure S4

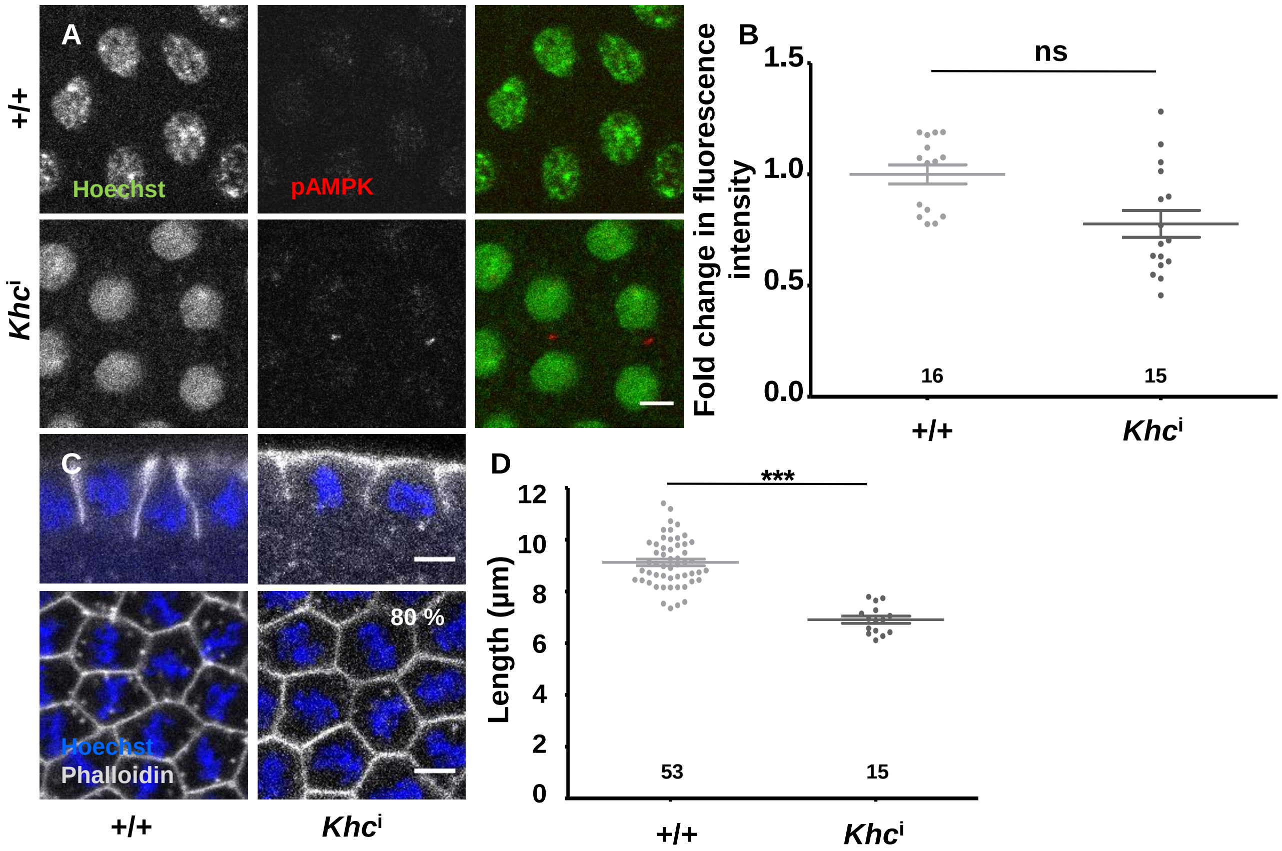

Chowdhary et. al. Figure S5
